# Opportunities and challenges for the development of “core outcome sets” in neuro-oncology

**DOI:** 10.1093/neuonc/noac062

**Published:** 2022-03-14

**Authors:** Christopher P Millward, Terri S Armstrong, Heather Barrington, Andrew R Brodbelt, Helen Bulbeck, Anthony Byrne, Linda Dirven, Carrol Gamble, Paul L Grundy, Abdurrahman I Islim, Mohsen Javadpour, Sumirat M Keshwara, Sandhya T Krishna, Conor L Mallucci, Anthony G Marson, Michael W McDermott, Torstein R Meling, Kathy Oliver, Barry Pizer, Puneet Plaha, Matthias Preusser, Thomas Santarius, Nisaharan Srikandarajah, Martin J B Taphoorn, Colin Watts, Michael Weller, Paula R Williamson, Gelareh Zadeh, Amir H Zamanipoor Najafabadi, Michael D Jenkinson

**Affiliations:** Institute of Systems, Molecular, & Integrative Biology, University of Liverpool, Liverpool, UK; Department of Neurosurgery, The Walton Centre NHS Foundation Trust, Liverpool, UK; Neuro-Oncology Branch, Center for Cancer Research, National Cancer Institute, Bethesda, Maryland, USA; Institute of Population Health, University of Liverpool, Liverpool, UK; Institute of Systems, Molecular, & Integrative Biology, University of Liverpool, Liverpool, UK; Department of Neurosurgery, The Walton Centre NHS Foundation Trust, Liverpool, UK; Brainstrust—The Brain Cancer People, Cowes, UK; Department of Palliative Care, Cardiff and Vale UHB, Cardiff, UK; Marie Curie Research Centre, Cardiff University, Cardiff, UK; Department of Neurology, Leiden University Medical Center, Leiden, the Netherlands; Department of Neurology, Haaglanden Medical Center, The Hague, the Netherlands; Institute of Population Health, University of Liverpool, Liverpool, UK; Department of Neurosurgery, University Hospital Southampton, Southampton, UK; Institute of Systems, Molecular, & Integrative Biology, University of Liverpool, Liverpool, UK; Department of Neurosurgery, The Walton Centre NHS Foundation Trust, Liverpool, UK; National Centre for Neurosurgery, Beaumont Hospital, Dublin, Ireland; Institute of Systems, Molecular, & Integrative Biology, University of Liverpool, Liverpool, UK; Department of Neurosurgery, The Walton Centre NHS Foundation Trust, Liverpool, UK; Department of Neurosurgery. Alder Hey Children’s NHS Foundation Trust, Liverpool, UK; Department of Neurosurgery. Alder Hey Children’s NHS Foundation Trust, Liverpool, UK; Department of Neurology, The Walton Centre NHS Foundation Trust, Liverpool, UK; Division of Neuroscience, Florida International University, Florida, USA; Department of Neurosurgery, Geneva University Hospital, Geneva, Switzerland; International Brain Tumour Alliance, Surrey, UK; Institute of Life Course and Medical Sciences, University of Liverpool, Liverpool, UK; Nuffield Department of Surgical Sciences, University of Oxford, Oxford, UK; Division of Oncology, Department of Medicine I, Medical University of Vienna, Vienna, Austria; Department of Neurosurgery, Addenbrooke’s Hospital & University of Cambridge, Cambridge, UK; Institute of Systems, Molecular, & Integrative Biology, University of Liverpool, Liverpool, UK; Department of Neurosurgery, The Walton Centre NHS Foundation Trust, Liverpool, UK; Department of Neurology, Leiden University Medical Center, Leiden, the Netherlands; Department of Neurology, Haaglanden Medical Center, The Hague, the Netherlands; Institute of Cancer and Genomic Sciences, University of Birmingham, Birmingham, UK; Department of Neurology, University Hospital and University of Zurich, Zürich, Switzerland; Institute of Population Health, University of Liverpool, Liverpool, UK; Department of Surgery, University of Toronto, Toronto, Canada; University Neurosurgical Center Holland, Leiden University Medical Centre, Haaglanden Medical Center, Haga Teaching Hospitals, Leiden and The Hague, the Netherlands; Institute of Systems, Molecular, & Integrative Biology, University of Liverpool, Liverpool, UK; Department of Neurosurgery, The Walton Centre NHS Foundation Trust, Liverpool, UK

**Keywords:** core outcome set, clinical trial, effectiveness, glioma, meningioma

## Abstract

Core Outcome Sets (COS) define *minimum* outcomes to be measured and reported in clinical effectiveness trials for a particular health condition/health area. Despite recognition as critical to clinical research design for other health areas, none have been developed for neuro-oncology. COS development projects should carefully consider: scope (how the COS should be used), stakeholders involved in development (including patients as both research partners and participants), and consensus methodologies used (typically a Delphi survey and consensus meeting), as well as dissemination plans. Developing COS for neuro-oncology is potentially challenging due to extensive tumor subclassification (including molecular stratification), different symptoms related to anatomical tumor location, and variation in treatment options. Development of a COS specific to tumor subtype, in a specific location, for a particular intervention may be too narrow and would be unlikely to be used. Equally, a COS that is applicable across a wider area of neuro-oncology may be too broad and therefore lack specificity. This review describes why and how a COS may be developed, and discusses challenges for their development, specific to neuro-oncology. The COS under development are briefly described, including: adult glioma, incidental/untreated meningioma, meningioma requiring intervention, and adverse events from surgical intervention for pediatric brain tumors.

Clinical trials investigate comparative effectiveness of therapeutic strategies to allow new treatment recommendations to be made.^1^ Comparative effectiveness is defined as the superiority or noninferiority of one therapeutic option in comparison to another. Evaluation requires investigators to choose “what” outcomes to measure, and a method “how” and time-point “when” for their measurement.^2^ Clinical trials are only as credible as their outcomes.^3^ Fundamentally, outcomes should measure treatment benefit and harm.^4^ However, outcome measurement across similar clinical trials in neuro-oncology is often inconsistent, selectively reported, and not always relevant to key stakeholders. This limits the ability to make judgments about comparative effectiveness, generates research waste, slows therapeutic progress, and diminishes the generosity of time and effort given by patients.

A Core Outcome Set (COS) is defined as *an agreed and standardized set of minimum outcomes that should be measured and reported in a clinical trial for a specific health condition/health area* and are increasingly recognized as critical to clinical research design.^5^ Implementing a COS does not preclude the measurement of additional outcomes. Using a COS enables judgment of comparative effectiveness across trials and facilitates data meta-analysis.^4^ To date over 370 COS have been developed.^6^

Multiple organizations support the use of COS in clinical trials, including the National Institute of Health Research (NIHR), the European Medicines Agency (EMA), WHO, and numerous patient organizations. COS are well established in other medical subspecialties. In rheumatology, the “Outcome Measures in Rheumatology” (OMERACT) initiative consists of 35 working groups across the spectrum of rheumatological disease, and has demonstrated uptake of COS in over 81% of rheumatoid arthritis clinical trials conducted between 2002 and 2016.^7^ Other well-established initiatives include “The Cochrane Skin—Core Outcome Set Initiative” (CS-COUSIN) and “Core Outcome Sets in Women’s and Newborn Health” (CROWN). No COS have been developed for neuro-oncology.

This review discusses outcomes in neuro-oncology clinical trials, issues to consider in COS development, and a summary of active neuro-oncology COS projects.

## Outcomes in Clinical Trials

Selection and measurement of appropriate outcomes in clinical trials is critical.^8^ A trial or study outcome is a measurable variable examined in response to a treatment or intervention, to assess effectiveness or harm. Traditional measures of response or time-dependent metrics are important (eg, radiological tumor response or survival), but are somewhat limited because they fail to characterize the functional or symptomatic effect of the tumor on the person. Outcomes should measure, either directly or indirectly, how patients feel, function, and survive.^9^ Patients want to live longer, but not necessarily at the expense of quality of life.^10^

The US Food & Drug Administration (FDA) describe four categories of clinical outcome assessment (COA): patient-reported (eg, health-related quality of life by questionnaire), clinician-reported (eg, performance status), observer-reported (eg, informal caregivers), or performance outcomes (eg, neurocognitive tests).^11^ Brain tumor clinical trials increasingly include the measurement of patient-reported outcomes, but the level of reporting may be suboptimal.^12,13^

## Identifying “Need” for COS in Neuro-Oncology

COS should only be developed for neuro-oncology if there is a clearly identified need and future uptake is anticipated. Examples of need include standardizing outcomes to allow meta-analysis and generation of new knowledge, or identifying outcomes of core importance to patients that are not currently measured in clinical trials—a scenario which may result in treatment recommendations that are not acceptable to patients.^4^ COS are increasingly being developed for routine practice which may also justify need.^6^ Future uptake requires broad engagement of healthcare professionals conducting neuro-oncology research. The COS should be widely disseminated through conference presentations, publications, and communication with policy makers, charities, and patient organizations.^2^

The Core Outcome Measures in Effectiveness Trials (COMET) initiative brings together people interested in the development and application of COS.^14^ New COS projects should be registered, and if the same COS are listed as under development by another research group or if overlap exists, COMET facilitates communication between research groups to promote collaboration and prevent duplication of effort and research waste.^14^

The existence of a COS with similar scope to one planned does not constitute an absolute contraindication to its development, and may be beneficial within neuro-oncology. Consider the hypothetical situation of a disease-specific COS being developed for pediatric medulloblastoma, and a researcher developing a broader COS defining outcome measures for clinical trials of surgically managed posterior fossa tumors. The disease-specific COS may include outcomes highly relevant to medulloblastoma key stakeholders, eg, disease-specific treatments such as adjuvant chemoradiotherapy. However, a broader COS that reflects surgical intervention for a particular anatomical location (ie, posterior fossa), will identify adverse events associated with surgery that will be relevant to other key stakeholders, including those invested in medulloblastoma. The combination of a disease-specific and treatment-specific COS will cover outcomes of relevance to all key stakeholders.

## Considerations for a Neuro-Oncology COS

### Standards for COS Development

The COMET Handbook^2^ illustrates methodological considerations for COS development.^14^ The Core Outcome Set Standards for Development (COS-STAD) is the product of a consensus process from an international panel of COS experts.^15^ Eleven minimum standards provide a framework of issues to consider. A study protocol should be published a priori describing the COS under development, according to the 13 minimum Core Outcome Set-Standardized Protocol Items (COS-STAP).^16^

### COS Development Process

COS development starts by identifying and extracting outcomes verbatim, from published and ongoing clinical studies.^2^ These are grouped, deduplicated, and classified into “unique” outcomes.^8^ This list is supplemented with patient-centered outcomes, for example, through semistructured interviews with patients with lived experience of the disease.^2^ The importance of each unique outcome is rated by stakeholder participants, for instance, via a Delphi-consensus process. The language used should be understandable by all. A priori description of the scoring process and definitions of consensus should be described.^2^ After 2 or more rounds, agreement on some outcomes which are of critical importance may be achieved. A consensus meeting of key stakeholders is held to resolve outcomes where a decision has not been made.^2^

For patients with brain tumors, neurologic symptoms such as impaired communication or cognitive function, might affect participation, and semistructured interviews, Delphi surveys, and consensus meetings should be appropriately adapted. Including patient research partners in all aspects of the COS study will generate solutions to ensure meaningful participation.^17,18^

### Scope of a COS

Scope defines COS usage including setting (clinical trials and/or routine practice), health condition/s, population/s and intervention/s. Failure to establish clarity of scope can restrict future uptake. Establishing scope within neuro-oncology poses a challenge, since CNS tumors are heterogeneous with respect to histopathology and molecular subtypes, are anatomically distributed, and subject to variable treatment strategies.

A highly specific COS for right frontal lobe glioblastoma would have a clear and unambiguous application. Development would include patient stakeholders with similar lived experiences, and the consensus process would have fewer conflicting opinions on what outcomes should be considered core. However, future uptake would be very limited—since researchers would not undertake clinical trials exclusively for right frontal lobe glioblastoma.

Alternatively, a broad COS for all CNS tumors would have extensive uptake. However, the development process would likely result in highly polarized opinions on the importance of individual outcomes, and only those that are broad reaching would achieve consensus, eg, quality of life, progression, and survival.

The scope of a COS for neuro-oncology should strike the balance between specificity (to ensure relevance) and applicability (to prevent research waste) and must carefully consider disease, anatomic, symptom, and treatment factors. Importantly, stakeholders should discuss and agree scope in advance of COS development.

### Stakeholders Involved in COS Development

COS development should include all key stakeholders (including patients) with the condition or their representatives, researchers who will use the COS in the future, and healthcare professionals with experience of caring for patients.

Patient involvement has been defined as “research being carried out ‘with’ or ‘by’ members of the public rather than ‘to’, ‘about’ or ‘for’ them”.^19^ Patients are key participants in COS development.^15^ Consider that seizures are a core outcome, is frequency or severity more important? Only through patient participation can this opinion be obtained to ensure that resulting treatment recommendations are patient-centered. Patients should also be involved as research partners to shape the design, conduct, and dissemination of the research.^2,17,18^ By involving brain tumor patients as research partners, people with lived experience can help to identify potential challenges for other brain tumor patient participants. They can advise on ways to support patient participants who might have difficulty completing a Delphi survey on a computer due to visual difficulties, be limited in their ability to concentrate due to fatigue, or struggle at a consensus meeting if they have physical, communication, or cognitive impairments. Achieving consensus can be difficult when patients have very different lived experiences, for example the priorities of an eloquent versus noneloquent anatomical location. Facilitation of the consensus meeting with patient input would balance polarized opinions and reaffirm the goal. To not include patient participants with a wide variety of challenges would deny agency to the range of challenges faced by brain tumor patients and could render the COS unrepresentative of the wider population. Evidence shows that patient input to COS development is increasing year-on-year.^6^ Added value also comes from incorporating the views of patient representatives (eg, family members, support workers, charities) as both patient research partners and patient participants. Some of the impacts from brain tumors may be more apparent to a carer than the patient.

Researchers who will use the COS in future trials, and healthcare professionals with experience of caring for patients should also be included. The neuro-oncology tumor board is expansive, and so it is important to seek participation from as many representative groups as possible, as early as possible at both the study advisory level (in the early phase of a project) and participant level. Importantly, brain tumor clinical trials are often multi-center, and multi-national and input should be balanced geographically amongst those who will use the COS. Cultural and language barriers should be considered during COS development.

### Dissemination and Uptake of COS

There is little point in developing a COS if researchers do not use it when designing new clinical trials, or as one patient poignantly said “core outcome sets are far too important to sit on a shelf gathering dust”.^20^ Dissemination of a COS is critical to uptake, but other barriers to uptake also exist including lack of validated measures, lack of patient and other key stakeholder involvement, and a lack of awareness of the COS.^21^ COS should be published and freely available, and clearly describe the scope and development process. COMET have produced the Core Outcome Set-STAndards for Reporting: The COS-STAR Statement to facilitate this aim.^22^

## Neuro-Oncology COS in Development

Four neuro-oncology COS projects are currently listed on the COMET registry as “*in development*” ^14^ and summarized in Table 1 and below.

**Table 1:** Core Outcome Set Standards for Development (COS-STAD) Recommendations Applied to Ongoing Neuro-Oncology COS Projects

Domain	Standard	Methodology	The COBra Study^a^	COSMIC: Intervention^b^	COSMIC: Observation^b^	The COMBAT Project^c^
**Scope** **specification**	1	The research or practice setting(s) in which the COS is to be applied	Later phase clinical effectiveness trials that will inform clinical decision making.	Later phase clinical effectiveness trials that will inform clinical decision making.	Clinical studies of incidental, minimally symptomatic, and untreated intracranial meningioma that will inform clinical decision making.	Routine clinical practice, individual surgeon and institution outcome reporting, and later phase clinical effectiveness trials that will inform clinical decision making.
	2	The health condition(s) covered by the COS	Intracranial glioma, both low- and high-grade.	Treated intracranial meningioma.	Incidental, minimally symptomatic, and untreated intracranial meningioma.	Primary pediatric CNS tumors observed in the pediatric population.
	3	The population(s) covered by the COS	Human adults aged 18 or above.	Human adults aged 18 or above.	Human adults aged 18 or above.	Human children aged 18 or below (although some pediatric neuro-oncology trials include patients beyond the age of 18).
	4	The intervention(s) covered by the COS	Interventions, alone or in combination, aimed at the tumor or direct effects of the tumor to include surgery, systemic anti-cancer treatments (including chemotherapy, immunotherapy and targeted therapies), radiotherapy and supportive interventions.	Interventions including surgical resection, radiotherapy, stereotactic radiosurgery, pharmacotherapy, perioperative care and supportive treatments; any of which may be in isolation or in combination with each other.	Active monitoring only as an intervention, but not treatment for an intracranial meningioma.	Interventions limited to surgical biopsy and/or surgical resection.
**Stakeholders** **involved**	5	Those who will use the COS in research/clinical practice	Clinical trialists and academic clinicians undertaking glioma trials.	Clinical trialists investigating interventions for intracranial meningioma.	Clinical trialists investigating active monitoring strategies for patients with intracranial meningioma.	Individuals, institutions, and policy makers evaluating patient outcomes, and clinical trialists investigating interventions for primary pediatric CNS tumors.
	6	Healthcare professionals with experience of patients with the condition	Multi-professional clinicians involved in glioma patient care; clinical trialists and academics involved in glioma research; policy makers and regulators.	This will include clinicians from multiple subspecialties and nonclinician healthcare professionals with active involvement in the care of patients with intracranial meningioma.	This will include clinicians from multiple subspecialties and nonclinician healthcare professionals with active involvement in the care of patients with intracranial meningioma.	This will include clinicians from multiple subspecialties and nonclinician healthcare professionals with active involvement in the care of patients with primary pediatric CNS tumors.
	7	Patients with the condition or their representatives	Persons with the condition and their family members will be included.	Patients with a diagnosis of intracranial meningioma who have received treatment will be included, along with relatives and carers of such patients.	Patients with a diagnosis of incidental intracranial meningioma who have not received treatment will be included, along with relatives and carers of such patients.	Patients with a diagnosis of a primary pediatric CNS tumor who have received surgical biopsy and/or surgical resection will be included, along with relatives and carers of such patients.
**Consensus** **process**	8	The initial list of outcomes considered both healthcare professionals’ and patients’ views.	A trial registry search and systematic literature review of glioma trial outcomes will consider healthcare professionals’ views. A systematic review of qualitative literature will identify patient and key stakeholder perceptions of important outcomes. Semistructured interviews with patients’ and caregivers will identify additional outcomes of importance based on “lived experience”.	A trial registry search and systematic literature review of intracranial meningioma trial outcomes will consider healthcare professionals’ views, whilst Patient Research Partner input and published semistructured interviews with patients will consider patients’ views.	A trial registry search and systematic literature review of clinical studies of incidental, minimally symptomatic, and untreated intracranial meningioma will consider healthcare professionals’ views, whilst Patient Research Partner input and published semistructured interviews with patients will consider patients’ views.	A trial registry search and systematic literature review of primary pediatricCNS tumor trial outcomes will consider healthcare professionals’ views, whilst a systematic literature review of patient-reported outcomes will consider patients’ views. Semistructured interviews with patients and/or their relatives and carers will contribute to the long-list.
	9	A scoring process and consensus definition were described a priori.	Participants will rate each of the outcomes on a 9-point Likert scale, (1–3, not important; 4–6, important but not critical; and 7–9, important and critical. During Round 1, participants can add outcomes they feel are missing. Votes from individuals in each stakeholder group will be given equal weighting. All original outcomes will be presented in Round 2. Outcomes added by participants in Round 1 will be presented in Round 2. In Round 2, respondents will be presented with their own rating for each outcome and how it was rated by their own stakeholder group. Based on this information, respondents will be invited to amend their score, if they wish. During Round 2, participants can rate the outcomes suggested in Round 1.	Outcomes to be scored on a 9-point Likert scale, whereby(1–3) is of limited importance, (4–6) is important but not critical, and (7–9) is critically important. Consensus defined as 80% or more of participants scoring an outcome as critical (7–9).	Outcomes to be scored on a 9-point Likert scale, whereby(1–3) is of limited importance, (4–6) is important but not critical, and (7–9) is critically important. Consensus defined as 80% or more of participants scoring an outcome as critical (7–9).	To be determined, protocol in development.
	10	Criteria for including, dropping, adding outcomes were described a priori.	The threshold for consensus for inclusion in or exclusion from the COS will be ≥ 70%, informed by those used in comparable COS development studies. After the Delphi, outcomes will be proposed for inclusion in the final COS if ≥ 70% respondents rate the item as 7–9 and ≤ 15% rate the item as 1–3. Items will be proposed for exclusion from the final COS if ≥ 70% respondents rate the item as 1–3 and ≤ 15% rate the item as 7–9. Those outcomes that do not reach agreement after the two Delphi rounds will be discussed in the consensus meeting, together with the items proposed for inclusion and exclusion.	Critical outcomes will be included.Outcomes rated as critical by only 50% or less of participants would be dropped. Outcomes to be discussed and voted on at the consensus meetings will be those that are neither included or dropped.	Critical outcomes will be included.Outcomes rated as critical by only 50% or less of participants would be dropped. Outcomes to be discussed and voted on at the consensus meetings will be those that are neither included or dropped.	To be determined, protocol in development.
	11	Care was taken to avoid ambiguity of language used in the list of outcomes.	Both study content and study materials will utilize plain language summaries and clinical explanations where necessary. All materials will be reviewed with Patient Research Partners and pilot tested with patients and healthcare professionals.	Both study content and study materials will utilize plain language summaries and clinical explanations where necessary. All materials will be reviewed with Patient Research Partners and pilot tested with patients and healthcare professionals.	Both study content and study materials will utilize plain language summaries and clinical explanations where necessary. All materials will be reviewed with Patient Research Partners and pilot tested with patients and healthcare professionals.	Both study content and study materials will utilize plain language summaries and clinical explanations where necessary. All materials will be reviewed with Patient Research Partners and pilot tested with patients and healthcare professionals.

(a) The COBra Study. COMET registration No. 1793, accessible at www.comet-initiative.org/Studies/Details/1793, and is also registered on PROSPERO (CRD42021236979). This study has Cardiff University sponsorship and Cardiff University School of Medicine Research Ethics Committee approval (Ref SMREC 21/59), and is funded by The Brain Tumour Charity (GN-000704). (b) The COSMIC Project. COMET registration No. 1508, accessible at www.comet-initiative.org/Studies/Details/1508. This study has University of Liverpool sponsorship and ethical approval (Ref UoL001601), and is funded by The Brain Tumour Charity (Jxr30103). (c) The COMBAT Project. COMET registration No. 1968, accessible at www.comet-initiative.org/Studies/Details/1968. Sponsorship, ethical approval, and funding are yet to be obtained for this project.

### The COBra Study—Development of a Core Outcome Set and Identification of Patient-Reportable Outcomes for Primary Brain Tumor Trials

Use of consistent outcomes in glioma trials that allow holistic analysis of treatment benefits can underpin informed care decisions, and there has been an increasing emphasis on quality, alongside length, of survival.^10^ When assessing treatments, PROs (as described above) capture participants’ own insight into the impact of treatment on wellbeing. This provides a perspective beyond assessment of disease control and survival gains which can more fully inform future patients’ treatment choices.^10^ At present, outcome assessment in glioma trials is inconsistent, preventing evidence synthesis across studies and limiting change to clinical practice. The COBra Study aims to develop a COS for use in glioma trials which will be applicable across glioma types, with identification of subsets as required. Due to the interest in core PROs in cancer, the secondary aim is to identify the COS outcomes which can be patient-reported.

Further information is available at https://www.cardiff.ac.uk/marie-curie-palliative-care-research-centre/research/research-portfolio/cobra.

### The COSMIC Project—Core Outcome Sets for Meningioma In Clinical Studies

Meningioma are the commonest primary brain tumor and are a highly heterogeneous disease entity.^23^ Symptomatic and/or critically located meningioma often require surgical resection. Radiotherapy may be used as primary treatment and for residual, recurrent, or inoperable disease. No effective pharmacotherapy exists.^24^ Incidental and minimally symptomatic meningioma may never require treatment, but are usually subject to interval MRI monitoring to monitor growth.^24^

Meningioma clinical effectiveness trials are sparce,^25–31^ and clinical studies of incidental/untreated meningioma rare,^32,33^ but important research questions need to be answered, especially for recurrent, clinically-aggressive and incidental meningioma. Currently, the outcomes measured and described in meningioma clinical studies are highly heterogeneous and there are likely to be fundamental differences between the outcomes considered core by key stakeholders from these two patient cohorts. The COSMIC Project (www.thecosmicproject.org) will establish the *minimum* outcomes that should be reported in meningioma clinical effectiveness trials (COSMIC: Intervention) and studies of incidental/untreated meningioma (COSMIC: Observation).

### The COMBAT Project—Core Post Operative Morbidity Set for Paediatric BrAin Tumors

CNS tumors are the most common solid tumor in those aged 0–19 years, represent 6% across all ages, and are the most common cause of cancer death in this population.^23^ Pediatric brain tumors are associated with high morbidity which may have lifelong consequences for survivors, both from the disease and treatment. Classifying and reporting postoperative morbidity in pediatric brain tumors is challenging. The disease area is highly heterogeneous, anatomically distributed, and associated with location-specific morbidity. Pathology may also dictate the aggressiveness of surgical intent and the level of postoperative morbidity which is acceptable to achieve adequate disease control. In addition, presurgical neurological condition and co-morbidity status can be variable at diagnosis and may contribute to cumulative postoperative morbidity. Finally, many children will go on to have systemic therapies or radiotherapy which also affect tumor-associated morbidity. Transparent and reproducible morbidity reporting helps to manage patient and parent expectations, provides a standardized way to compare adverse events in clinical or research studies and provides a benchmark to compare clinical services. The application of existing morbidity tools to report pediatric brain tumor surgery harms is inadequate.^34^ The COMBAT project will develop a core set of adverse outcomes for children undergoing tumor biopsy and/or resection which are determined to be of importance to all key stakeholders.

## Conclusions

COS have not yet been developed for neuro-oncology but could facilitate the harmonization of outcome measurement across clinical studies. However, a standardized approach to statistical analysis, interpretation, and reporting of outcomes is also required to ensure results are valuable for clinical decision making. In addition, the use of COS ensures that outcomes of relevance and importance to patients are evaluated in clinical studies conducted for their benefit. COS development projects across the breadth of malignant, nonmalignant, and pediatric neuro-oncology have commenced. Clinical triallists should be encouraged to develop COS for use in future neuro-oncology clinical studies when one does not exist, or use a developed COS as a minimum, when available. The uptake and impact of COS in neuro-oncology clinical studies should be assessed in the future.
